# Stabilization of Ferroelectric Phase in Highly Oriented Quinuclidinium Perrhenate (HQReO_4_) Thin Films

**DOI:** 10.3390/ma14092126

**Published:** 2021-04-22

**Authors:** Junyoung Lee, Woojun Seol, Gopinathan Anoop, Shibnath Samanta, Sanjith Unithrattil, Dante Ahn, Woochul Kim, Gunyoung Jung, Jiyoung Jo

**Affiliations:** School of Materials Science and Engineering, Gwangju Institute of Science and Technology, Gwangju 61005, Korea; jyl@gist.ac.kr (J.L.); wooj0210@gist.ac.kr (W.S.); anoopnemom@gmail.com (G.A.); shibnaths2@gmail.com (S.S.); Sanjith123@gmail.com (S.U.); danteahn95@gm.gist.ac.kr (D.A.); kwc0714@gist.ac.kr (W.K.); gyjung@gist.ac.kr (G.J.)

**Keywords:** quinuclidinium perrhenate, plastic crystals, molecular ferroelectric crystals, ferroelectricity

## Abstract

The low-temperature processability of molecular ferroelectric (FE) crystals makes them a potential alternative for perovskite oxide-based ferroelectric thin films. Quinuclidinium perrhenate (HQReO_4_) is one such molecular FE crystal that exhibits ferroelectricity when crystallized in an intermediate temperature phase (ITP). However, bulk HQReO_4_ crystals exhibit ferroelectricity only for a narrow temperature window (22 K), above and below which the polar phase transforms to a non-FE phase. The FE phase or ITP of HQReO_4_ should be stabilized in a much wider temperature range for practical applications. Here, to stabilize the FE phase (ITP) in a wider temperature range, highly oriented thin films of HQReO_4_ were prepared using a simple solution process. A slow evaporation method was adapted for drying the HQReO_4_ thin films to control the morphology and the temperature window. The temperature window of the intermediate temperature FE phase was successfully widened up to 35 K by merely varying the film drying temperature between 333 and 353 K. The strategy of stabilizing the FE phase in a wider temperature range can be adapted to other molecular FE materials to realize flexible electronic devices.

## 1. Introduction

Plastic molecular crystals exhibiting switchable polarization are a unique class of ferroelectric (FE) materials owing to their environmental friendliness, structural tunability, and mechanical flexibility [1,2,3,4,5,6,7,8,9,10]. Compared to inorganic FE materials, the low-temperature processibility of plastic crystals makes it an attractive choice for FE applications [9,11,12,13,14,15,16,17,18,19,20,21,22]. The major drawback of molecular ferroelectric crystals are their low spontaneous polarization, low melting temperature, low Curie temperature, and small dielectric constant, which needs to be improved for replacing it with the inorganic FE materials [23]. Several molecular FE compounds have been investigated; however, many practical challenges exist—such as controlling crystal orientations and polarization directions, making it difficult to manipulate the FE properties [8,12,13,14,15,18,21,23]. When compared to bulk FE crystals, inorganic FE thin films have a much broader impact and applications in microelectronics [24]. It is highly challenging to realize ferroelectricity in molecular thin-films because most of the molecular ferroelectrics are monopolar axial. Proper control of crystal orientation is required for molecular crystals to exhibit switchable polarization [8]. Several experimental challenges have arisen because to maximize the polarization, the thin films should be grown with a specific crystallographic orientation. Recently, ferroelectricity has been observed in several plastic crystals that can overcome this issue of uniaxial polarization in molecular ferroelectric crystals [13,21]. The high-temperature paraelectric phase of plastic crystals have cubic symmetry similarly to the inorganic perovskites [13]. The high-temperature phase also possesses more than three polarization axes, making it suitable to grow FE thin films with switchable polarization at low temperatures [13]. Recently, quinuclidine perrhenate (HQReO_4_), a molecular FE crystal with a polarization axis that can be rotated through the rotation of ionic molecules in the crystal, has been investigated for FE applications [2,5,13,25,26]. HQReO_4_ crystallizes in three phases, namely room temperature phase (RTP), intermediate temperature phase (ITP), and high-temperature phase (HTP) [2]. The HTP is plastic, and when cooled down to ITP, the FE polarization can be controlled in the as-grown crystals of HQReO_4_ [2]. The RTP and ITP phases are non-centrosymmetric. The RTP crystallizes in the orthorhombic crystal system with a space group Pmn_21_ [2]. The FE phase is ITP, which is only stable for a narrow temperature window, ranging from 345 to 367 K, above and below which no ferroelectricity is observed in bulk crystals [2]. For standard electronic devices, the operating temperature range is 0–343 K, and specialized electronic devices that require a higher temperature (e.g., military use) need to operate in the temperature range of 218 to 398 K [1,2,3]. Thus, for practical applications, the required temperature window of 0–398 K should be considered. Therefore, the FE material should exhibit stable ferroelectricity and operate under a wider temperature window. However, in the case of the HQReO_4_ ITP, the range is very small (345–367 K), which is even narrower than the conventional temperature range of electronic devices. Therefore, it should be widened for practical applications.

Growing thin films of HQReO_4_ has been suggested as one feasible way to expand the narrow temperature window of ITP [5,13]. Compared with bulk crystals, thin films are widely used in the FE industry due to the flexibility and ease of processability [13]. Moreover, the temperature window in which ITP is stabilized can be controlled by varying the thin film deposition parameters or post-deposition annealing conditions such as annealing temperature/duration. In this study, we have drop-casted thin films of HQReO_4_ on ITO/glass substrates and widened the temperature window of ITP by controlling the drying temperature. A slow evaporation technique was adapted to control the morphology and microstructure of the thin films. By controlling the drying temperature during the slow evaporation, we achieved a wide temperature window of the ITP phase up to 35 K compared to 22 K in HQReO_4_ bulk crystals.

## 2. Materials and Methods

### 2.1. Preparation of Quinuclidine Perrhenate Thin Films

The thin films were prepared using the solution process. First, 1-azabicyclo octane [2.2.2]/quinuclidine (C_7_H_13_N) and perrhenic acid (HO_4_Re) solution (75–80 wt. % in water) were thoroughly mixed at 500 rpm using a stirrer for 1 h. Water was used as the solvent. For thin film preparation, 0.2 mL of the solution was drop cast onto ITO/glass substrates. The ITO/glass substrates were washed using acetone, ethanol, isopropyl alcohol before deposition of the films. A slow evaporation method was used to dry the water solvent. The sample was placed in a vacuum oven until the water is dried. Several samples were prepared by varying the drying temperature. The samples that dried at 60, 70, and 80 °C are labeled as QP-60, QP-70, and QP-80. A higher drying temperature results in a faster evaporation rate, which seriously impacts the crystalline quality and roughness of the as-grown films. The XRD spectra of QP-95 film, prepared at 95 °C, exhibits inferior crystalline quality, as shown in Figure S1. We could not obtain any FE characteristics from this film. Hence, the drying temperatures were chosen to facilitate slow evaporation. The drying time was fixed at 4 h for all the samples. The top electrode Au was deposited using the electron beam evaporation technique with a shadow mask. 

### 2.2. Characterizations

The polarization–voltage (P-V) hysteresis loops of fabricated Au/HQReO_4_/ITO capacitor were measured using a FE tester (Radiant Technology, Albuquerque, NM, USA). The surface morphology and cross-sectional images of HQReO_4_ thin films were observed using field emission scanning electron microscopy (FE-SEM) (S-4700, Hitachi, Tokyo, Japan). From the cross-sectional SEM images, the thickness of our samples was estimated to be around 2.5 ± 0.25 μm. Atomic force microscopy (AFM) (XE 100, Park systems, Suwon, Korea) was used to measure the surface roughness of the films. The crystalline structure of the films was analyzed using an X-ray diffractometer with Cu Kα radiation (Bruker, D8 Advance, Bruker, Billerica, MA, USA). The XRD patterns were recorded at various temperatures to observe the structural variations in HQReO_4_ thin films. The dielectric constant was measured using Keysight E4980A LCR Meter (Keysight technologies, Santa Rosa, CA, USA) (@ 100 KHz).

## 3. Results and Discussions

**Figure 1:**
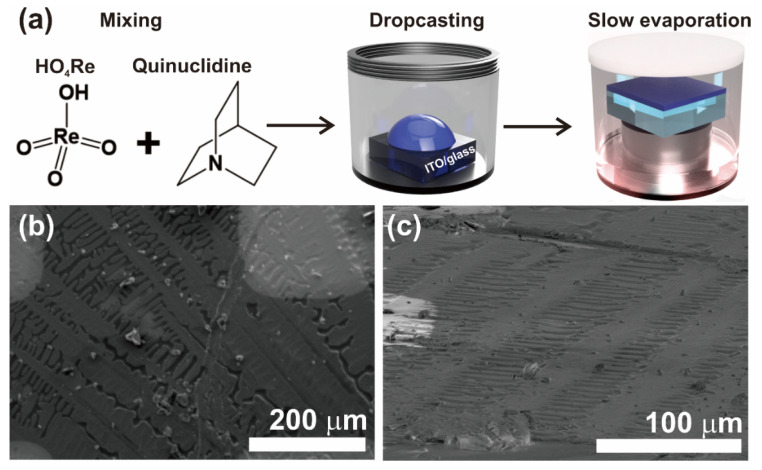
(**a**) A schematic showing the experimental procedure, with (**b**) top-view and (**c**) tilted-view SEM images of QP-80.

A schematic showing the preparation of HQReO_4_ thin films is shown in Figure 1a. The thin films were prepared using the solution process. The solution was drop-casted onto ITO/glass substrate, followed by a slow evaporation technique. For slow evaporation, the HQReO_4_/ITO/glass film was kept on an elevated platform with a small amount of water in the vial. The slow evaporation technique was adapted in our study because fast evaporation using hotplate led to inferior crystalline and FE characteristics.

**Figure 2:**
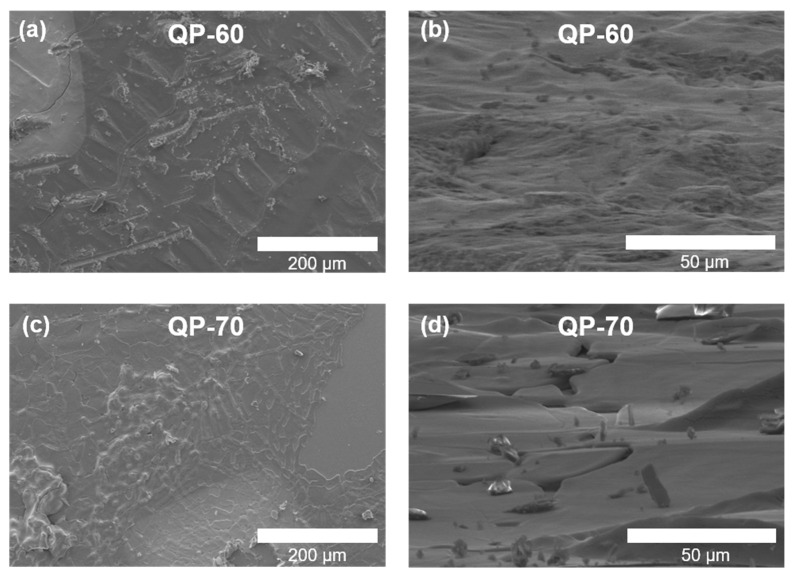
SEM images of (**a**,**b**) QP-60 and (**c**,**d**) QP-70 thin films.

The surface morphology of the QP-80 sample observed using SEM is shown in Figure 1b,c. Figure 2 shows the SEM images of QP-60 and QP-70 samples. The cross-sectional images of the films are shown in Figure S2. A drastic change in the surface morphology is observed with an increase in the drying temperature. The QP-60 and QP-70 samples exhibit inferior morphology and microstructure to the QP-80 film. The microstructure and morphology have a direct impact on the FE properties.

**Figure 3:**
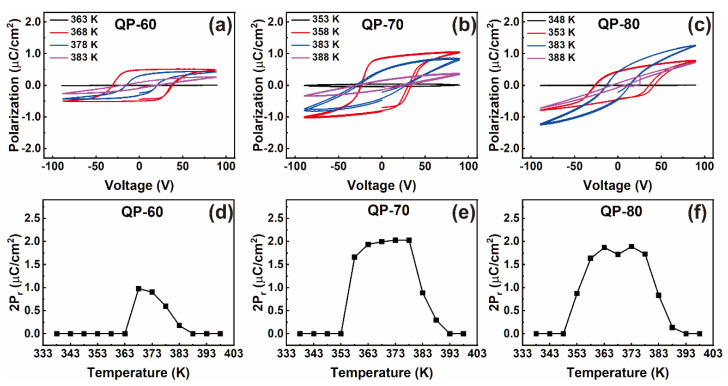
(**a**–**c**) The temperature-dependent P-V loops and (**d**–**f**) variation of 2*P_r_* values with the temperature of QP-60, QP-70, and QP-80 capacitors.

The FE properties of these Au/HQReO_4_/ITO capacitors are investigated by recording the P-V loops. As the FE phase formation of HQReO_4_ films depends on the ambient temperature, the temperature-dependent P-V loops are measured and scrutinized. The P-V loops of QP-60, QP-70, and QP-80 capacitors recorded at several temperatures are studied and are shown in Figure 3a–c. The P-V loops were measured in the temperature range from 338 to 398 K at intervals of 5 K. Note that only the P-V loops recorded at specific temperatures are shown in Figure 3a–c. The FE characteristics were temperature-dependent, and the nature of this dependence is strongly subjected to the drying temperature. The nature of temperature dependence is summarized in terms of remnant polarization (2P_r_), as shown in Figure 3d–f.

There is a specific temperature range in which the samples behave as FE and exhibit typical FE hysteresis. As the temperature increases, the samples transforms to a polar phase at a specific temperature. Upon further increase beyond a certain temperature, the phase once again transforms to a non-polar phase. Thus, the temperature window in which the FE behavior exists is controlled by varying the drying temperature. The temperature window of the polar FE phase in the QP-60 sample is only 15 K (368–383 K). In contrast, the QP-70 sample has a corresponding window of 30 K (358–388 K). The QP-80 sample showed FE stability for a temperature window of 35 K range from 353 to 388 K, which is nearly 1.6 times higher than the temperature window (22 K) of the ITP in bulk crystal. It is worth mentioning that the drying temperature can directly control the stability of ITP in a wider temperature range. In some FE materials, the volume of pores or physical defect concentration changes the intensity of the internal stress. The change in internal stress causes changes in the phase transition temperature [27]. In our films, a dramatic change in microstructure with a change in drying temperature is evidenced. Thus, a similar effect can be expected, which causes the widening of the polar phase temperature window.

The 2P_r_ values are also found to vary with the drying temperature. The QP-70 sample exhibited the highest 2P_r_ value of 2 μC/cm^2^. Note that the 2P_r_ values are much lower than the reported values in bulk, which can be correlated with the inferior surface morphology or microstructure of our thin films. The SEM images shown in Figure 2 indicate a rough surface for the QP-60 sample, which improved with the drying temperatures. The QP-80 sample shows better surface morphology; however, it is still not a smooth surface. There are several visible cracks on the surface, making the sample leaky. The roughness of the films was measured using AFM (Figure S3). All samples show high roughness values (>20 nm). Such high roughness values create discontinuities between the microcrystallites and make the capacitor a complex assembly of microcapacitors. Thereby, the complex combination turns the film far away from the electrically continuous medium. Thus, though each microcrystal may have sufficient polarization, the macroscopic polarization is low due to discontinuities. In conventional FE materials, the effect of defects (discontinuities, porosity etc.) on electric properties is well known. 

The temperature-dependent dielectric constant-voltage curves of QP-60, QP-70, and QP-80 capacitors are shown in Figure S3. As shown in Figure S3, when the ITP volume fraction in the films increases, the films turn FE and exhibit butterfly curves. 

**Figure 4:**
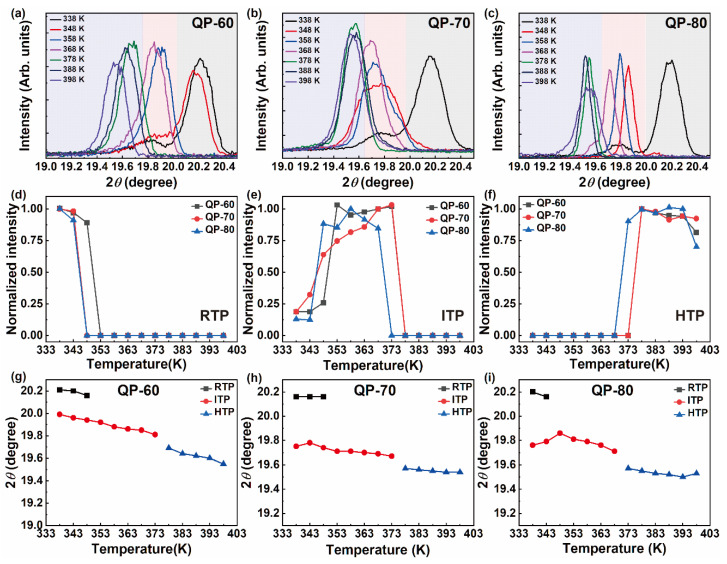
(**a**–**c**) The temperature-dependent XRD patterns of QP-60, QP-70, and QP-80 thin films. (**d**–**f**) The corresponding intensity variations of RTP, ITP, and HTP, respectively. (**g**–**i**) The temperature-dependent 2θ variations in QP-60, QP-70, QP-80 thin films.

To investigate the correlation between the variation observed in the temperature window and the crystal structure, a detailed temperature-dependent XRD analysis was carried out. The XRD spectra of QP-60, QP-70, and QP-80 films are shown in Figure 4a–c.

The intensity variations of RTP, ITP, and HTP with temperature are shown in Figure 4d–f, respectively. Figure 4g–i show the shift in 2θ variations with temperature. Note that the phase transformation occurs with an increase in temperature, and the crystal structure changes from orthorhombic (RTP) to rhombohedral (ITP) and then to cubic (HTP). To check the crystalline nature of the films, the XRD patterns were recorded in a wide range (2θ = 10–50°) at room temperature (RTP). The corresponding spectra of the QP-60 sample are shown in Figure S5, matching well with the reported pattern [2]. It is worth mentioning that all the thin films are highly oriented. The peak observed near 2θ = 20° corresponds to the reflection from the (111) plane of the orthorhombic RTP. In ITP and HTP, the reflection is from (10−1) and (110) planes, respectively. To further confirm the crystalline quality and orientation, reciprocal space maps around the (10−1)/(110) reflection of the QP-60 sample were also recorded at various temperatures (348–393 K), as shown in Figure S6. The RSMs appear as distinct spots. The temperature-dependent reciprocal space maps shown in Figure S6 confirm the highly oriented nature of the as-grown films. The splitting of the peaks and the shift in the peak positions with an increase in temperature are also clearly visible in the RSMs. 

With an increase in the temperature, the 2θ values shift to lower values, suggesting an increase in the d-spacing and cell volume. When the phase transforms from RTP to HTP, the cell volume increases. The ITP crystallizes in a rhombohedral structure with a larger cell volume than RTP, whereas the cubic HTP has the highest cell volume. The reflections correspond to (10−1) plane in rhombohedral ITP and (110) plane in cubic HTP. The shift in 2θ values is also gradual, indicating the thermal energy modifies the crystal structure from RTP to HTP through ITP. Notably, the temperature window of the ITP has increased with the drying temperature. For the QP-80 sample, the intensity of RTP drops to zero at a temperature of 348 K, indicating the phase transforms to ITP, while for QP-60 and QP-70 samples, the RTP is stable up to 353 K. Thus, from the XRD analyses, it is confirmed that phase transformation and phase stabilization window varies with the drying temperature.

**Figure 5:**
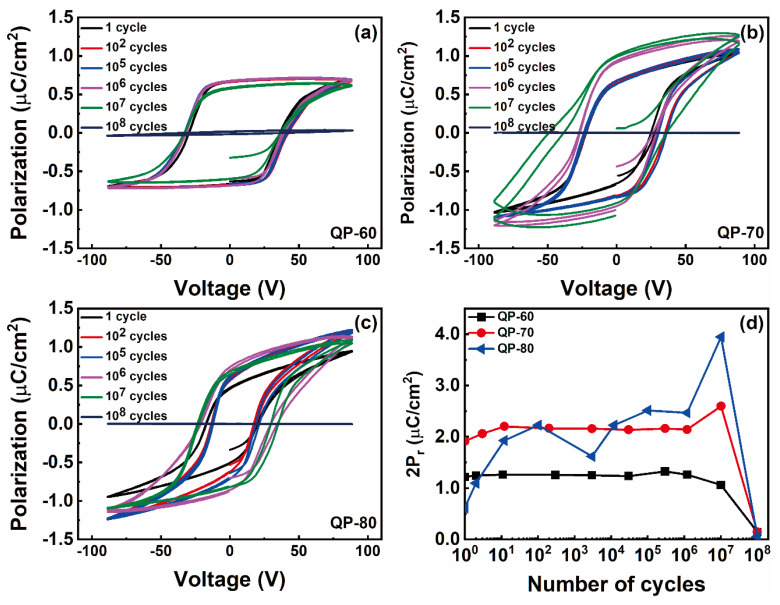
Field cycling and fatigue response for (**a**) Au/QP-60/ITO, (**b**) Au/QP-70/ITO, and (**c**) Au/QP-80/ITO capacitors. (**d**) The variation in 2P_r_ values with the electric field cycling.

The field cycling stability and retention were tested up to 10^8^ cycles for all the samples. Figure 5a–c shows the field cycling performance of QP-60, QP-70, and QP-80 samples, respectively. The variation in 2P_r_ values with field cycling is shown in Figure 5d. The capacitors exhibit leaky behavior with field cycling; notably, the QP-80 sample exhibits more leaky behavior than QP-60 and QP-70 capacitors.

The origin of fatigue in organic FE capacitors is different from inorganic capacitors. In P(VDF-TrFE) capacitors, the fatigue is attributed to delamination of the top Au electrode that occurs from the gases formed during the phase decomposition during field cycling [28]. Even though molecular FE material-based capacitors have a different origin of polarization, the phase decomposition can be one reason for the observed fatigue. Another reason can be permanent phase transformation to HTP with the constant application of the electric field. Especially for Au/QP-80/ITO capacitors, the fatigue is much higher than the other capacitors, indicating the films formed at higher drying temperatures exhibit fatigue at a lower number of field cycling. However, detailed analysis is required to identify the reason for fatigue in our QP capacitors. Note that the polarization was retained for 10^7^ cycles, which is much higher than the other polymer FE materials, and can find applications in reliable FE electronic devices.

## 4. Conclusions

The temperature-dependent FE properties of HQReO_4_ thin films deposited using the solution process were investigated in detail. The thin films were deposited by varying the drying temperature—60, 70, and 80 °C. The FE characteristics, phase transformation between RTP, ITP, and HTP, and surface morphology were significantly dependent on the film drying temperature. XRD analyses revealed the highly oriented nature of the as-grown thin films, which was independent of the drying temperature. The QP-80 sample exhibited better surface morphology than QP-60 and QP-70 films. The phase transformations between RTP, ITP, and HTP were observed from the temperature-dependent XRD analysis, which was highly dependent on the drying temperature. The temperature window in which ITP is stabilized was successfully widened by controlling the drying temperature. A 2P_r_ value up to 2 μC/cm^2^ was achieved for the QP-70 capacitor. The fatigue analyses revealed that the fabricated capacitors exhibit stable polarization values up to 10^7^ cycles. The low-temperature processability of HQReO_4_ thin films is highly beneficial for fabricating low-cost, flexible electronic devices. The strategy to control the temperature window of the ITP in HQReO_4_ by merely varying the drying temperature can be applied to other molecular FE thin films.

## Data Availability

Data are contained within the article.

## Supplementary Materials

The following are available online at https://www.mdpi.com/article/10.3390/ma14092126/s1. Figure S1: XRD spectra of QP-95 thin film. Figure S2: The cross-sectional SEM images of QP-60 and QP-80 thin films. Figure S3: AFM images of QP-60, QP-70, and QP-80 samples. Figure S4: Dielectric constant vs voltage plots of (a) QP-60, (b) QP-70, and (c)QP-80 capacitors at a frequency of 100 KHz. (d–f) the corresponding variation of dielectric constant (@ 50 V) with temperature. Figure S5: The XRD patterns of QP-60 sample measured at room temperature. Figure S6: The temperature-dependent reciprocal space maps (RSM) around the (10-1) plane of ITP and (110) plane of HTP.
