# Supplementary material for: Stabilization of Ferroelectric Phase in Highly Oriented Quinuclidinium Perrhenate (HQReO4) Thin Films
Source: Materials (Basel). 2021 Apr 22;14(9):2126. doi: 10.3390/ma14092126 (PMC8122725; doi:10.3390/ma14092126)
Supplement: Supplementary file 1 [file materials-14-02126-s001.zip › materials-1171174-supplementary.pdf]

Supplementary Information

Stabilization of ferroelectric phase in highly oriented quinuclidinium perrhenate ($HQReO_4$) thin films

Jun Young Lee, WooJun Seol, Gopinathan Anoop, Shibnath Samanta, Sanjith Unithrattil, Dante Ahn, Woochul Kim, Gun Young Jung, and Ji Young Jo*

School of Materials Science and Engineering, Gwangju Institute of Science and Technology, Gwangju 61005, South Korea.
*Email: jyjo@gist.ac.kr

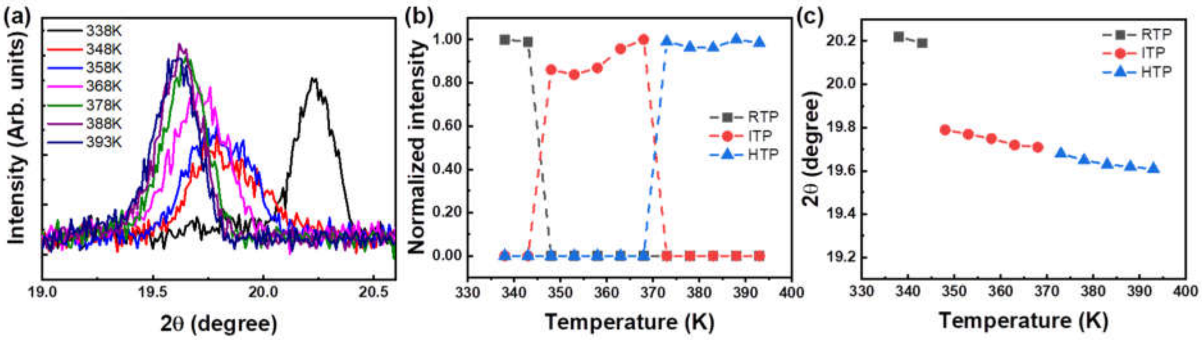

Figure S1. (a) The temperature-dependent XRD spectra of the QP-95 sample. The variation of (b) RTP, ITP and HTP intensities and (c) 2θ positions with temperature.

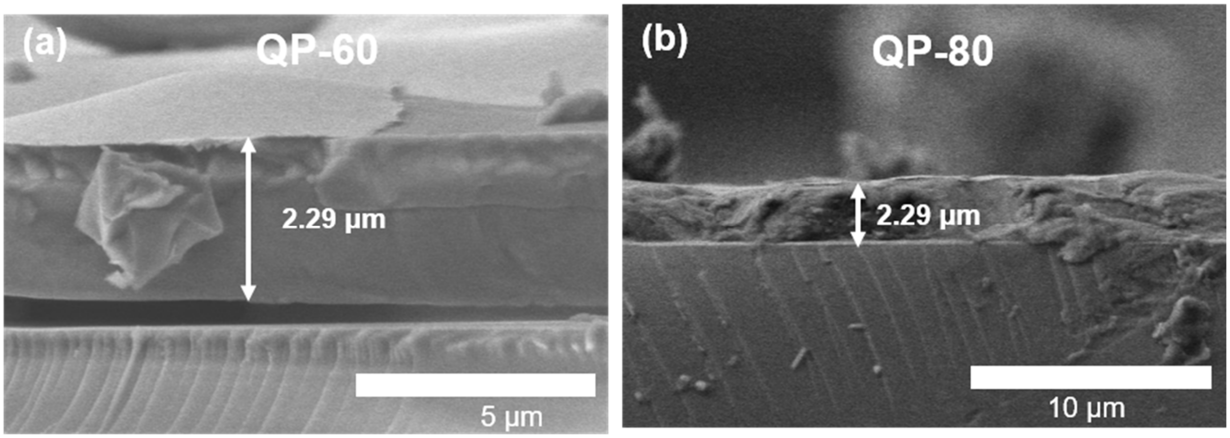

Figure S2. The cross-sectional SEM images of QP-60 and QP-80 thin films. The films exhibit a thickness of around 2.3 µm.

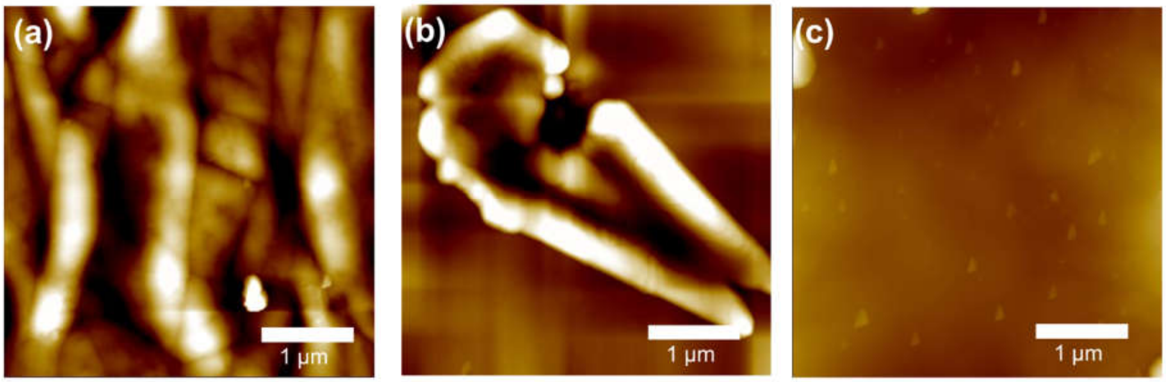

Figure S3. AFM images of (a) QP-60, (b) QP-70, and (c) QP-80 films. The RMS roughness (Ra) of the films were 24.3, 19.8, and 22 nm for QP-60, QP-70, and QP-80 samples, respectively.

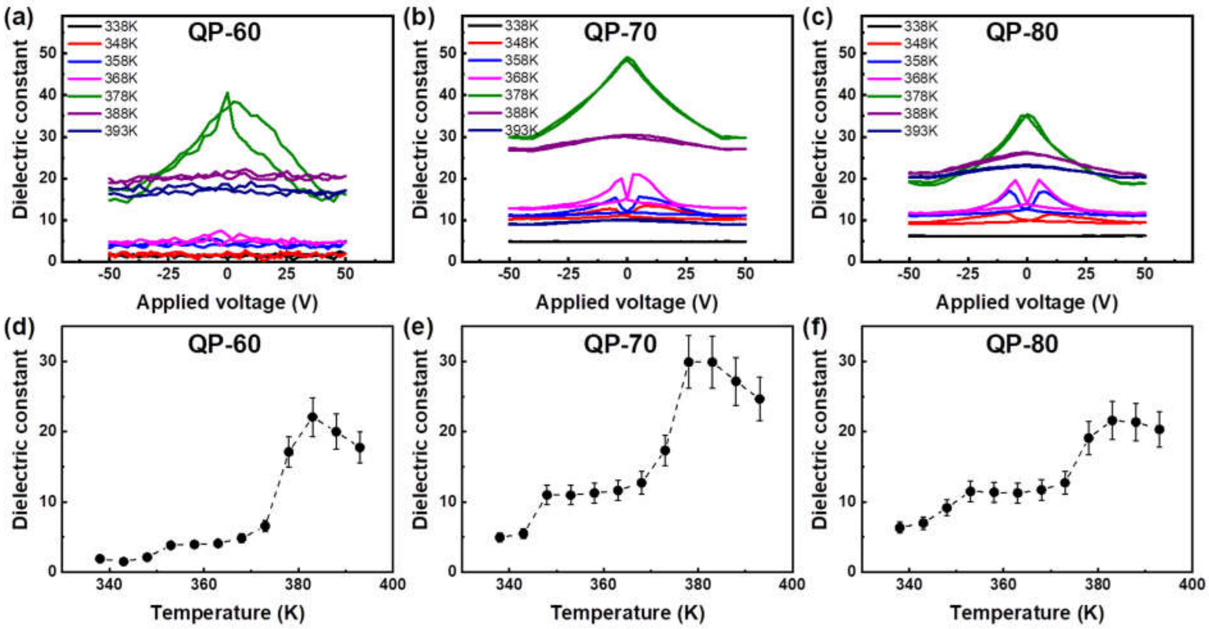

Figure S4. Dielectric constant vs voltage plots of (a) QP-60, (b) QP-70, and (c)QP-80 capacitors at a frequency of 100 KHz. (d-f) the corresponding variation of dielectric constant (@ 50 V) with temperature.

The temperature-dependent dielectric constant-voltage curves of QP-60, QP-70, and QP-80 capacitors are shown in Fig. S4. The contribution from the hysteresis domain switching and the accumulation of depletion charges occur in ferroelectric thin films resulting in butterfly-shaped loops. The capacitance and the dielectric constant of a ferroelectric material vary because the domain switches between the two polarization states when the applied voltage is swept between negative and positive voltages. As shown in Fig. S4, when the ITP volume fraction in the films increases, the films turn FE and exhibit butterfly curves. The temperature window in which the films exhibit typical FE behavior varies with the drying temperature. The QP-80 capacitor exhibits FE characteristics for a widened temperature window compared to QP-60 and QP-70 capacitors.

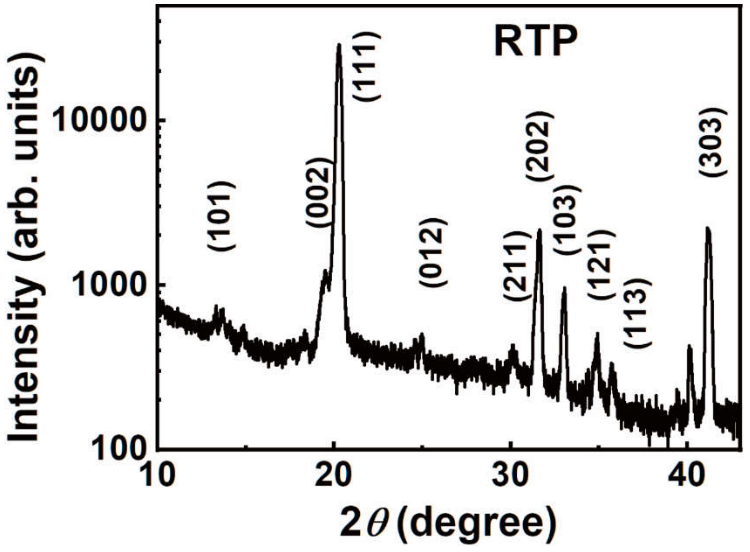

Figure S5. The XRD patterns of QP-60 sample measured at room temperature.

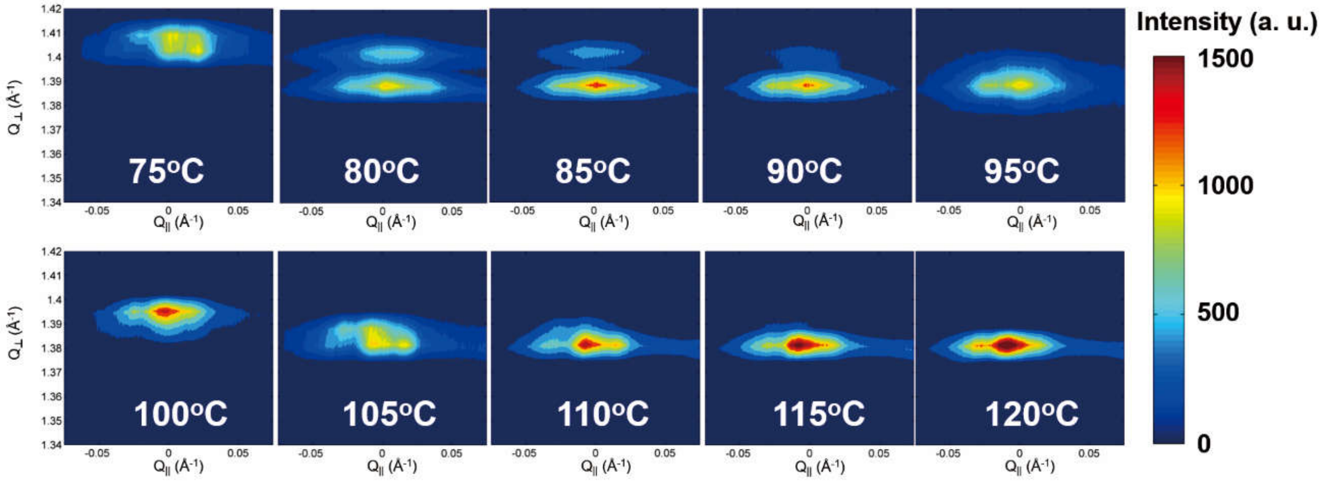

Figure S6. The temperature-dependent reciprocal space maps (RSM) around (10-1) plane of ITP and (110) plane of HTP.
